# 234. Dalbavancin versus Outpatient Parenteral Antimicrobial Therapy with Vancomycin for Treatment of Bone and Joint Infections in a Veteran Population

**DOI:** 10.1093/ofid/ofab466.436

**Published:** 2021-12-04

**Authors:** Emily A Gibbons, Teri L Hopkins, Manuel R Escobar, Linda Yang, Elizabeth Walter, Jose Cadena-Zuluaga

**Affiliations:** 1 South Texas Veterans Health Care System, San Antonio, Texas; 2 South Texas Veterans Health Care System, UT Health San Antonio, UT Austin College of Pharmacy, Baltimore, Maryland; 3 University of Texas health and science center San Antonio, Audie L. Murphy VA Medical Center, San Antonio, Texas

## Abstract

**Background:**

Dalbavancin is a long-acting lipoglycopeptide with broad gram-positive activity. A long half-life makes it an attractive treatment option for bone and joint infections (BJI). Previous studies have demonstrated efficacy of dalbavancin in the treatment of BJI. Based on these studies, our institution established a protocol for using dalbavancin as an alternative to IV antibiotics via PICC line.

**Methods:**

Chart review was performed to compare outcomes of patients who were treated with dalbavancin versus vancomycin for BJI from 8/2017 –7/2020. Patients that received two doses of dalbavancin for BJI were compared with patients who received OPAT with vancomycin during the same time period. Patients were excluded if they were bacteremic or received dalbavancin for another indication. Data was collected from the Veterans Health Administration’s Corporate Data Warehouse and retrospective chart review. No statistical analyses were performed due to the descriptive nature of this study.

**Results:**

A total of 59 patients were included; 25 received dalbavancin and 34 received vancomycin. Relevant differences in baseline characteristics included a higher proportion of patients with osteomyelitis (88% vs 74%) and refractory infection (64% vs 44%) in the dalbavancin group. More patients in the dalbavancin group (38% vs 24%) were readmitted for the same infection within one year, required (29% vs 21%) additional surgical intervention, and had increased CRPH on follow-up labs (32% vs 3%). Dalbavancin use likely expedited discharge in at least 5 cases where vancomycin levels were not therapeutic. No significant adverse effects due to dalbavancin were noted, aside from one patient with an increase in serum creatinine. In the vancomycin group, 8 patients changed antibiotics due to adverse effects or difficulty managing levels and 3 patients had ED visits for PICC line care.

**Conclusion:**

Dalbavancin may be a safe PICC-sparing treatment for BJI, particularly in cases where compliance is of concern, or there are logistical or tolerability issues with vancomycin. Our findings do raise concern for worse outcomes with dalbavancin, but the small sample size, difference in baseline characteristics between groups and descriptive nature of the study preclude any conclusions from being drawn.

**Disclosures:**

**All Authors**: No reported disclosures

